# Relationship between the components of mare breast milk and foal gut microbiome: shaping gut microbiome development after birth

**DOI:** 10.1080/01652176.2024.2349948

**Published:** 2024-05-10

**Authors:** Eman A. Mady, Haruna Osuga, Haruka Toyama, Hussein M. El-Husseiny, Ryo Inoue, Harutaka Murase, Yuki Yamamoto, Kentaro Nagaoka

**Affiliations:** a Laboratory of Veterinary Physiology, Department of Veterinary Medicine, Tokyo University of Agriculture and Technology, Tokyo, Japan; b Department of Animal Hygiene, Behavior, and Management, Faculty of Veterinary Medicine, Benha University, Moshtohor, Toukh, Elqaliobiya, Egypt; c Laboratory of Veterinary Surgery, Department of Veterinary Medicine, Tokyo University of Agriculture and Technology, Tokyo, Japan; d Laboratory of Animal Science, Department of Applied Biological Science, Setsunan University, Osaka, Japan; e Hidaka Training and Research Center, Japan Racing Association, Hokkaido, Japan

**Keywords:** Horses, gut bacteria, breast milk, metabolomics

## Abstract

The gut microbiota (GM) is essential for mammalian health. Although the association between infant GM and breast milk (BM) composition has been well established in humans, such a relationship has not been investigated in horses. Hence, this study was conducted to analyze the GM formation of foals during lactation and determine the presence of low-molecular-weight metabolites in mares’ BM and their role in shaping foals’ GM. The fecal and BM samples from six pairs of foals and mares were subjected to 16S ribosomal RNA metagenomic and metabolomic analyses, respectively. The composition of foal GM changed during lactation time; hierarchical cluster analysis divided the fetal GM into three groups corresponding to different time points in foal development. The level of most metabolites in milk decreased over time with increasing milk yield, while threonic acid and ascorbic acid increased. Further analyses revealed gut bacteria that correlated with changes in milk metabolites; for instance, there was a positive correlation between *Bacteroidaceae* in the foal’s gut microbiota and serine/glycine in the mother’s milk. These findings help improve the rearing environment of lactating horses and establish artificial feeding methods for foals.

## Introduction

The gut microbiota (GM), composed of bacteria in the digestive tract, plays a pivotal role in several physiological and immunological processes (Dougal et al. 2014; Husso et al. 2020). Disruptions in early bacterial colonization during infancy can increase vulnerability to various illnesses afterward in life (Kauter et al. 2019). Factors such as mode of birth (Dominguez-Bello et al. 2010), antibiotic administration (Zeissig and Blumberg 2014), environment of care (Brooks et al. 2014), and nutritional exposure, particularly breastfeeding (Ardeshir et al. 2014), can influence the acquisition of the intestinal microbiome (Mady et al. 2023).

Horses (Equus caballus) are hindgut fermenters with complex microbiota, primarily composed of anaerobic microbes that help digest high-fiber diets (Julliand et al. 2001; Husso et al. 2020; Zhu et al. 2021). GM is important in herbivore nutrition: it enables the breakdown of plant fibers into volatile fatty acids—the primary energy source of herbivores. The dietary requirements of foals change as they grow because of the changes in the milk components from mares and the transition to a solid diet. Correspondingly, the GM undergoes synchronization, favoring bacterial populations that more efficiently utilize the current diet of foals (De La Torre et al. 2019). The GM is not only essential to obtain nutrients but also to maintain health, modulate the immune system, and shelter against pathogenic microbes (Mousquer et al. 2021). Thus, disturbance of the GM of horses is associated with various diseases (Garber et al. 2020), including colitis (Harlow et al. 2013), laminitis (Moreau et al. 2014), and foal heat diarrhea or transient diarrhea in young foals (Kuhl et al. 2011). Hence, our perception of the colonization and development of the GM of horses should be improved. Since the composition of GM differs among animal species (Kobayashi et al. 2020), their development in each animal species should be examined (Collado et al. 2016). Even though a substantial portion of the microorganisms that will make up the foals’ GM is acquired at birth, early microbial colonization in horses occurs from the intrauterine stage (Mousquer et al. 2021). *Proteobacteria, Bacteroidetes, Actinobacteria, and Firmicutes* are the most abundant phyla at this stage. They are obtained from a mixture of bacteria found in the vagina, feces, and other maternal surroundings. During the first few weeks of life following birth, the GM of foals undergo progressive modification as a result of a variety of factors, including coprophagy, exposure to various settings, food composition, and medication use (Danel et al. 2013; Jacquay 2017; Pyles et al. 2023). The foals ‘ GM stabilizes during the first and second months of life.

Breast milk (BM) intake during infancy is crucial for optimal colonization and maturation of the infant microbiota. Milk compounds, such as oligosaccharides and hydrogen peroxide, promote the growth of dominant bacteria such as *Lactobacillus* and *Bifidobacterium* in humans and mice, respectively (Yatsunenko et al. 2012; Matsuki et al. 2016; Shigeno et al. 2019). These lactic acid bacteria elicit positive effects on the GM and are commonly used as probiotics for their health benefits (Chassaing et al. 2014; Kambe et al. 2020). Although the relationship between the development of GM in infants and BM is well established in humans (Bhinder et al. 2017; He et al. 2019), such a relationship has not been investigated in horses. The main objectives of this investigation were to analyze the formation of the GM in foals during lactation and to determine the presence of small-molecule metabolites related to foal development in mare BM. This study’s findings contribute to improving the breeding environment of lactating horses.

## Materials and methods

### Sample collection

**Figure 1.**
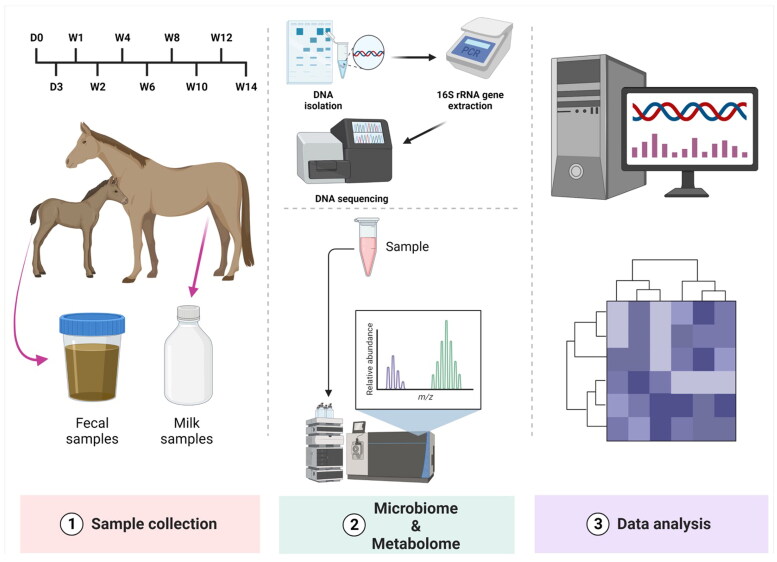
Schematic illustration of the present study design.

This study included six healthy thoroughbred adult pregnant mares (average age: 6 years) and their six foals at the JRA Hidaka Training Center. Fecal and BM samples were collected on Days 0 and 3 and Weeks 1, 2, 4, 6, 8, 10, 12, and 14 following the deliveries (Figure 1). The foal group consisted of four males and two females. None of the horses exhibited illnesses that required treatment throughout the sample collection period. Care of the horses was following international guidelines. Sample collections were performed at the Japan Racing Association (JRA) Hidaka Training and Research Center. The procedures described were approved by the Animal Care and Use Ethical Committee of Tokyo University of Agriculture and Technology (approval number: R03-177).

Fresh fecal samples were obtained from the foals’ and mothers’ rectums every morning. Samples of BM were gathered from the mothers. The collected fecal and BM samples were immediately stored at −80 °C. During the sample collection, each mother and foal initially grazed in a paddock until the foals turned 7 days old. After seven days, they were allowed to graze with other mothers and foals in the pasture. Initially, the grazing hours ranged from 8:00 to 15:30. When the foals were two months old, the grazing hours were adjusted from 13:00 to 8:00 the following day. During summer, grazing hours began at 15:30 and continued until 10:30 the next day. The grazing hours were modified to prevent the foals’ exposure to excessive heat and horseflies in the daytime.

The mothers had free access to grass as their primary feed. The feeding regimen included 1.5 kg of Stamm 30 (Hallway Feeds, Lexington, KY, USA) and 3 kg of swallows during the first month. Approximately two months after delivery, when the grazing hours were adjusted, the mares were fed 4, 2, and 1.5 kg of swallow, lusan, and Stamm 30, respectively. One month later, the feeding quantities were adjusted to 2.5, 2, and 1.5 kg of swallow, lusan, and Stamm 30, respectively. Two-month-old foals were gradually introduced to Stamm 30, starting with small amounts and gradually increasing to 1 kg.

### DNA extraction from feces and microbiome

The fecal samples were analyzed through 16S rRNA metagenomic analysis as described before (Kojima et al. 2022). Genomic DNA was extracted from the collected fecal samples using the Quick Gene DNA Tissue Kit S (Kurabo Industries, Osaka, Japan). A pre-trained QIIME2-compatible SILVA version 138 database (99% full-length sequences) was used to assign taxonomy to the filtered ASVs. All 16S rRNA sequencing data are available under accession number PRJNA1006090.

### Preparation of milk samples and metabolomics

The whole-milk samples obtained from individual mares were centrifuged at 21,500 × g for 60 min at 4 °C, and the middle layer was collected as skimmed milk. Untargeted metabolome analysis was accomplished using gas chromatography-mass spectrophotometry (GC–MS), according to a previous report (Kojima et al. 2022).

### Statistical analysis

MicrobiomeAnalyst (https://www.microbiomeanalyst.ca/MicrobiomeAnalyst/home.xhtml) and MetaboAnalyst (http://www.metaboanalyst.ca/) were employed to analyze the fecal microbiome and mare milk, respectively.

In microbiome analysis, Chao1 and Shannon indices were used to assess fecal microbiome diversity. Principal coordinate analysis (PCoA) was used to analyze the β-diversity of the microbiome samples and was plotted using the Unweighted UniFrac Distance index and permutational multivariate analysis of variance. Hierarchical cluster analysis was achieved using the Ward method and Unweighted UniFrac Distance index to group similar data into clusters. Linear discriminant analysis effect size (LEfSe) was used to compare the microbiome among the different groups.

In metabolomic analysis, partial least-squares discriminant analysis (PLS-DA) were exploited to analyze the variation among different groups. Spearman’s correlation test by microbial-metabolomics using MicrobiomeAnalyst was employed to evaluate the link between the fecal microbiome and the milk metabolome. Differences at *p*** **<** **0.05 were considered statistically significant.

## Results

### Gut microbiome in the mothers and foals

**Figure 2.**
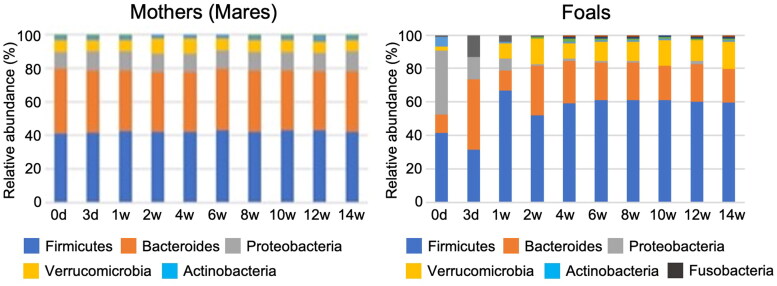
Relative abundance of bacterial phylum association with the mother (left side) and the foals (right side) during the lactation period (from day 3 to week 14 after birth).

**Figure 3.**
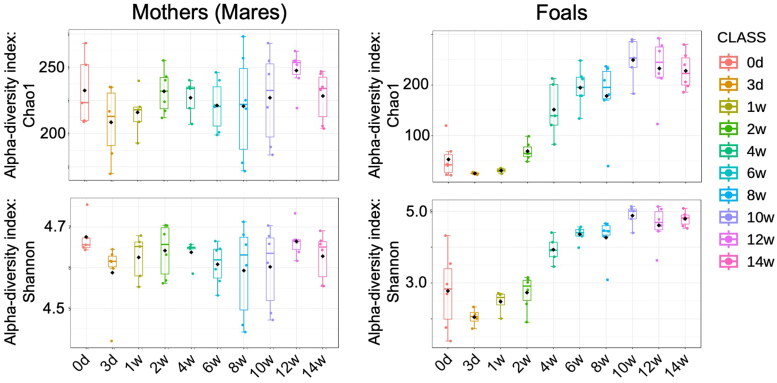
Alpha-diversity, measured by Chao1 and Shannon index is plotted for the mother (left side) and foals (right side) during the lactation period (from day 3 to week 14 after birth).

**Figure 4.**
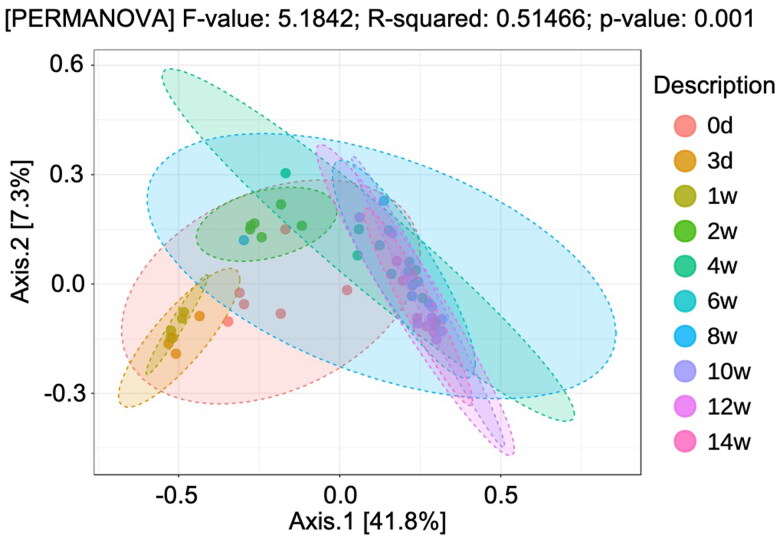
Plots of PCoA based on the the unweighted UniFrac distance index and permutational multivariate analysis of variance (*p*** **<** **0.05) during the lactation period (from day 3 to week 14 after birth).

The bacterial composition of the GM of the mothers and foals is shown at the phylum level (Figure 2). It predominantly consists of *Firmicutes*, *Bacteroides*, *Proteobacteria*, *Verrucomicrobia*, and *Actinobacteria*. Comparing mother and Foal, *Firmicutes* and *Verrucomicrobia* were more abundant in the foals than in the mothers. In contrast, *Bacteroides* and *Proteobacteria* were less abundant in the foals than in the mothers (Figure 2). Microbial α-diversity was stable among mothers across time (Figure 3). Among foals, Chao1, which is defined as “richness,” and Shannon, which is defined as “evenness” started to increase at one week after birth. Then, by eight weeks, the α-diversity of the foals was similar to that of the mothers (Figure 3). The different colors in Figure 4 represent the distinct clusters of the β-diversity of the GM of the foals from Day 0 to Week 14. This gradual temporal shift in the GM composition converged to a specific range approximately six weeks after birth (Figure 4).

### Hierarchical clusters in the gut microbiome of the foals

**Figure 5.**
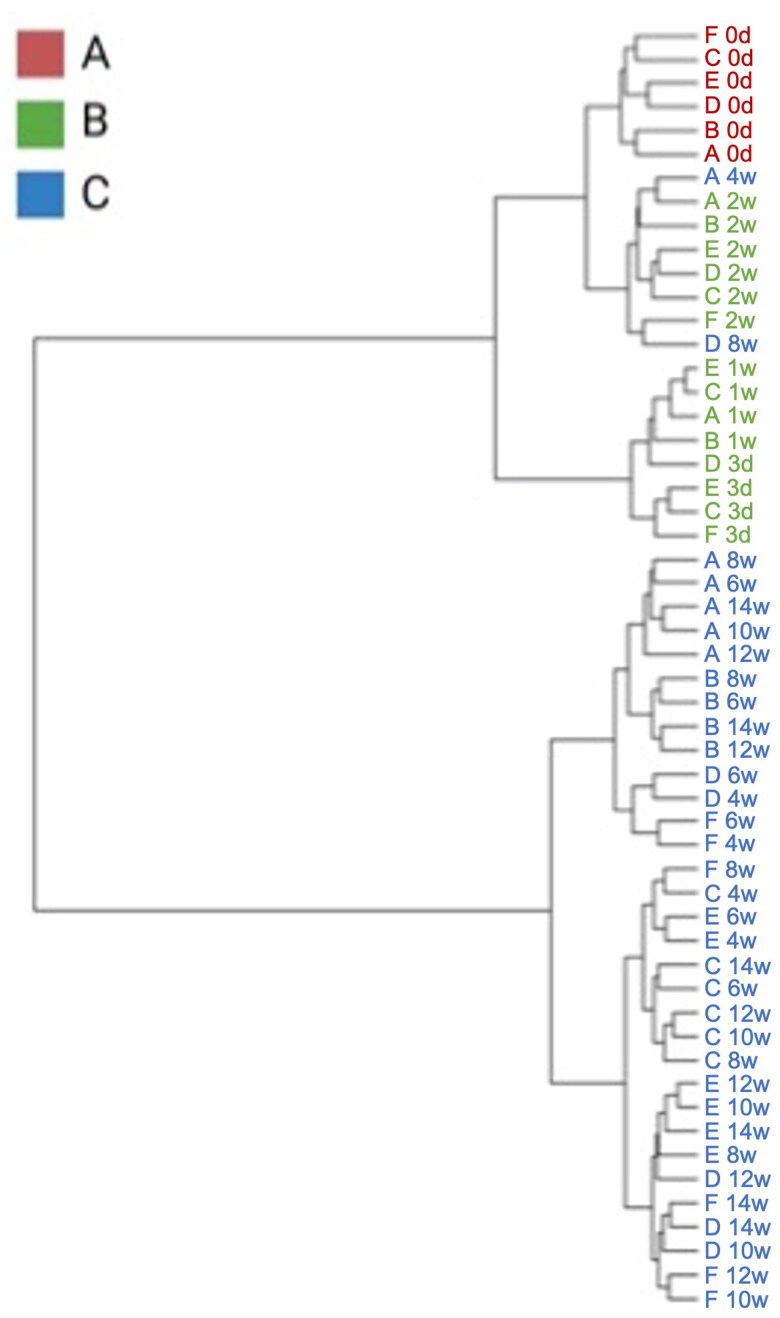
Dendrogram constructed with foals’ gut microbiome over experimental period by ward methods, based on the Unweighted UniFrac Distance index.

**Figure 6.**
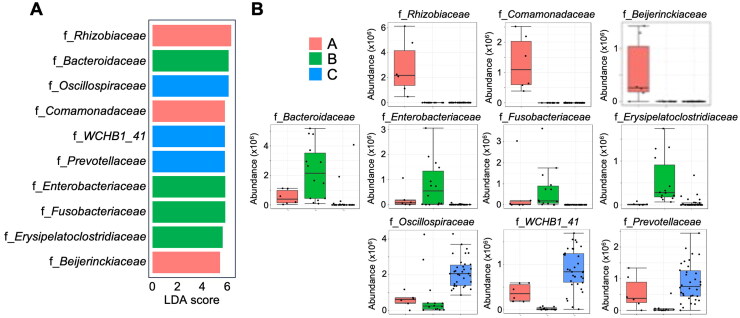
Composition of the foals’ gut microbiome at different time points. Linear discriminant analysis effect size (LEfSe analysis) at the family level (A) and changes in the abundance of each bacterium (B).

The GM of the foals was divided into three clusters: the day 0 sample is represented in red (Group A), the samples from 3 days to 2 weeks are represented in green (Group B), and the samples from 4 weeks to 14 weeks are represented in blue (Group C) colors (Figure 5). Subsequently, a LEfSe analysis was performed on these three groups (A, B, and C). The top 10 families attained from the LEfSe analysis at the family level (Figure 6A). The existence of Rhizobiaceae, Comamonadaceae, and Beijerinckiaceae characterized Group A. Group B ­consists of *Bacteroidaceae*, *Enterobacteriaceae*, *Fusobacteriaceae*, and *Erysipelatoclostridiaceae*. Group C consists of *Oscillospiraceae*, *WCHB1_41*, and *Prevotellaceae*. The changes in the abundance of each bacterium are shown in Figure 6B.

### Metabolites in breast milk

**Figure 7.**
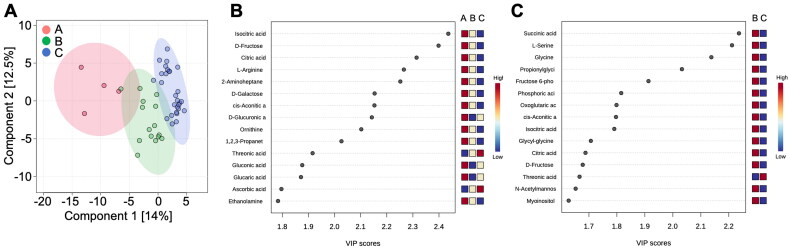
Variations in the metabolic components of the breast milk samples from the mares. A: 2D principal component analysis score plot of group A, group B, and group C. B: Important features identified by partial least-squares discriminant analysis among group A, group B, and group C. C: Important features identified by partial least-squares discriminant analysis among group B and group C. The colored boxes on the right indicate the relative concentrations of the corresponding metabolites in each group.

Untargeted metabolomic analysis revealed 131 metabolites in maternal BM samples. Similar to the GM analysis, groups A, B, and C represent day 0, day 3 to 2 weeks, and 4 to 14 weeks, respectively (Figure 7). Based on the PLS-DA results, metabolite composition of milk was shown to vary between groups (Figure 7A). Additionally, the top 15 substances in the three groups based on the VIP scores were presented (Figure 7B). The metabolite abundance declined from group A to group C, except for threonic acid and ascorbic acid, which increased over time (Figure 7B). On the other hand, the top 15 substances in groups B and C based on the VIP score are shown in Figure 7C. Most metabolite levels were lower in group C, with only threonine acid being higher in group C than in group B.

### Correlation between intestinal flora and components of breast milk

**Figure 8.**
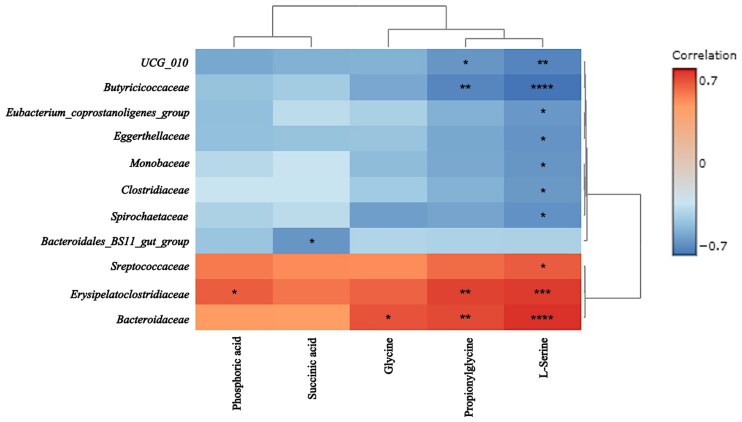
Heat map showing the correlation between the metabolome of the breast milk of mares and the development of the gut microbiome of foals. *p < 0.05, **p < 0.01, ***p < 0.001, and ****p < 0.0001.

The heat map illustrates the correlation between the metabolites level in the BM and the bacterium abundance in the GM of foals (Figure 8). Day 0 data were excluded from the correlation analysis because the colostrum might not yet influence the intestinal flora of the foals at that time point. The analysis revealed a significant positive link between Serine and *Streptococcaceae*, *Erysipelatoclostridiaceae*, and *Bacteroidaceae* (*R* = 0.66, 0.76, and 0.79, respectively; *p* = 0.042, 0.00038 and 0.00005, respectively). In contrast, a marked negative relationship was observed between Serine and *UCG_010*, *Butyricicoccaceae*, *Eubacterium_coprostanoligenes_group*, *Eggerthellaceae*, *Monoglobaceae*, *Clostridiaceae*, and *Spirochaetaceae* (*R* = −0.73, −0.79, −0.66, −0.68, −0.67, −0.66, and −0.69, respectively; *p* = 0.0017, 0.000036, 0.043, 0.018, 0.03, 0.04, and 0.013, respectively). Propionylglycine was significantly and directly correlated with *Erysipelatoclostridiaceae* and *Bacteroidaceae* (*R* = 0.74 and 0.72, respectively; *p* = 0.0012 and 0.0035, respectively), but significantly inversely correlated with *UCG_010* and *Butyricicoccaceae* (*R* = −0.67 and- 0.73, respectively; *p* = 0.03 and 0.0021, respectively). Glycine and *Bacteroidaceae* were significantly and positively correlated (*R* = 0.69, *p* = 0.013), as well as phosphoric acid and *Erysipelatoclostridiaceae* (*R* = 0.66, 0.76, *p* = 0.044). In contrast, succinic acid and *Bacteroidales_BS11_gut_group* were significantly and negatively correlated (*R* = −0.67, *p* = 0.03).

## Discussion

The gut microbiota (GM) plays an essential role in mammals, including horses, with diverse effects. In humans, extensive research has been conducted on the interaction between maternal breast milk (BM) components, GM formation, and infant intestinal microbiome development (Bhinder et al. 2017; He et al. 2019; Riederer et al. 2022). However, this relationship has not been investigated in horses. Therefore, this study aimed to investigate the formation of GM in foals, identify metabolites in the BM of mares, and examine the relationship between the two in detail. Similar to the findings of previous studies, *Firmicutes*, *Bacteroides*, *Proteobacteria*, and *Verrucomicrobia* were the dominant phyla in the GM of 1-week-old foals; thus, the composition of the GM of foals is consistent across different breeding environments (Costa et al. 2016; Schoster et al. 2016; 2017; Husso et al. 2020, O’Reilly et al. 2022). Furthermore, *Firmicutes*, *Bacteroidota*, and *Verrucomicrobiota* were dominant in both foals and mares; hence, these phyla constitute the core of the equine GM (Kauter et al. 2019).

Early GM formation in the foal is due to sudden postnatal exposure to environmental microorganisms, which elaborate the abundant bacterial diversity (Quercia et al. 2019; O’Reilly et al. 2022). In this study, Chao1 and Shannon indices, which exhibit α-diversity, showed that the foals were lower than their mothers up to 2 weeks of age but increased to values like their mothers by eight weeks. It was reported that the diversity of the intestinal flora in 60-day-old foals resembled that of adult mares (De La Torre et al. 2019). On the other hand, diarrhea is often prevalent in foals up to approximately four weeks of age, and the low alpha diversity observed during this period is thought to indicate an unstable gut flora (Rodriguez et al. 2015). Regarding β-diversity, we have clarified the change in the GM composition of the foal over time. Similar to α-diversity, the composition of the gut microbiota of the foals varied greatly from 3 days to 4 to 6 weeks of age, but a stable composition was observed after eight weeks of age.

Foals are mostly dependent on their dams during the first month of life since mother milk is their primary source of nutrition (Warren et al. 1998). Our metabolomic analysis showed that the levels of many metabolites in milk decreased during significant fluctuations in the foal’s GM. At the same time, milk production increases and elaborates the dilution of the metabolites observed in this study. Previous reports calculated that when milk intake is expressed as a percentage of body weight per day, foals consumed 27, 20, and 19.3% at ages 11, 25, and 39 days, respectively (Becvarova and Buechner-Maxwell 2012). It is suggested that milk containing higher level of metabolites may contribute to foals’ growth over the first month. The gradual switch from a milk diet to a solid diet enables the foals’ digestive systems to adapt gradually and makes it possible for the gut microbiota to establish, which is required for the digestion of fiber (Faubladier et al. 2014; Hausberger et al. 2015). According to the research done by Faubladier et al. foals can digest plant fibers by the time they are two months old.

Comparison of the foal microbiome at different time points revealed a developmental trajectory that gradually approached the typical adult configuration. The existing investigation found meaningful increases in *Rhizobiaceae*, *Comamonadaceae*, and *Beijerinckiaceae*, which are characteristic of GM at birth. These microbes are present in plant roots and other environmental sources. This suggests that these bacteria may have been acquired temporarily from the environment rather than in utero during pregnancy (Zakia et al. 2023). After birth, a variety of external routes of microbial transmission to the foal are possible, including ingestion of bacteria from the mother’s skin, udder, vagina, and hairs through licking the mother’s body (Garber et al. 2020). From 3 days to 2 weeks after birth, we identified characteristic families such as *Enterobacteriaceae*, *Bacteroidaceae, Fusobacteriaceae,* and *Erysipelato­clostridiaceae*. Consistent with this finding, the presence of these bacteria families has been reported in the intestines of 1–14 day old foals (Schoster et al. 2017). In addition, Quercia et al. said that the foal gut microbiota undergoes gradual changes from 3 days after birth, with the progressive acquisition of microorganisms typical of the milk community, such as *Enterococcus* and *Enterobacteriaceae* (Quercia et al. 2019). In early GM development, neonatal diarrhea is frequently observed in many animals, including foals (Frederick et al. 2009). In piglets, it has been reported that *Bacteroidetes* belonging to the *Bacteroidetaceae* are more common in healthy individuals than in those with diarrhea, while *Fusobacteriaceae* are more common in individuals with diarrhea (Yang et al. 2017; Gryaznova et al. 2022,). Although no diarrhea was observed in the foals in this study, the balance of these bacteria, especially higher *Bacteroidaceae*, during the GM development period may be involved in the growth of healthy foals.

A relationship between the BM of the mare and the development of the GM of the foal has been postulated. However, there have been few reports on identifying specific milk components and gut bacterial species and their relationship. This study identified serine and glycine as BM components positively correlated with *Bacteroidaceae*. These amino acids were reported to be more abundant in colostrum than in mature milk in humans, pigs, cows, and horses (colostrum was collected within two days of parturition in humans and one day in other animals; mature milk was collected 1-4 months after parturition in humans, three weeks after in pigs, two months after in cows, and one month after in horses (Davis et al. 1994)). Although it is not yet clear how serine and glycine in mother’s milk are involved in GM formation in the offspring, it has been reported that feeding these amino acids to piglets after weaning contributes to the development of the intestinal mucosa and intestinal barrier (Zhou et al. 2018; Ji et al. 2022). As mentioned above, *Bacteroidaceae* are abundant in the intestines of healthy piglets. It was also reported that *Bacteroidaceae* predominated the cecal bacterial community at day 7 of age in piglets, which can be related to their capability to ferment milk (Lerch et al. 2023). From these observations, it can be inferred that serine and glycine, which are abundant in mother’s milk, contribute to the health of foals from 3 days to 2 weeks of age by increasing *Bacteroidaceae* in the foal’s intestine and acting directly or indirectly on intestinal barrier formation.

In this study, metabolomic analysis of mare’s milk suggested the involvement of several metabolites in the formation of the gut microbiota of the foal. However, future studies may also benefit from considering metabolomic analysis of fecal samples to better understand the gut microbiota’s important role in producing specific metabolites. In addition, further studies are needed to validate the results of this study, such as measuring serine isomers (L-serine and D-serine) and increasing the number of samples, as well as to determine the direct relationship between serine/glycine and Bacteroidetes.

In conclusion, this study provides new insights into the relationship between the gut microbiota of foals and changes in the composition of the mare’s milk during lactation. There was a correlation between changes in the shape of the gut microbiota of the foal and changes in the composition of the breast milk of the mare. In particular, serine and glycine changes in the mother’s milk were suggested to be involved in the early development of the gut microbiome of the foal. These findings help improve the rearing environment of lactating horses and establish artificial feeding methods for foals.

## Data Availability

The datasets displayed in the present investigation can be found in online repositories. The repository/repositories’ names and the accession number(s) can be attained below: https://www.ncbi.nlm.nih.gov/genbank/, PRJNA1006090.
